# Toxicity and bioaccumulation of manganese and chromium in different organs of common carp (*Cyprinus carpio*) fish

**DOI:** 10.1016/j.toxrep.2021.02.003

**Published:** 2021-02-06

**Authors:** Zeeshan Ali, Ali Muhammad Yousafzai, Nadia Sher, Ijaz Muhammad, Gul E. Nayab, Syed Abdul Maajid Aqeel, Syed Touheed Shah, Michael Aschner, Ijaz Khan, Haroon Khan

**Affiliations:** a Department of Zoology, Islamia College University Peshawar, Pakistan; b Department of Chemistry, Islamia College University Peshawar, Pakistan; c Department of Zoology, Abdul Wali Khan University, Mardan, 23200, Pakistan; d Department of Molecular Pharmacology, Albert Einstein College of Medicine Forchheimer, 209 1300 Morris Park Avenue Bronx, NY, 10461, United States; e Department of Microbiology, Hazara University Mansehra, Pakistan; f Department of Pharmacy, Abdul Wali Khan University, Mardan, 23200, Pakistan

**Keywords:** HCT, Hematocrit, HGB, Hemoglobin, RBCs, Red Blood Cells, MCV, Mean corpuscular volume, MCH, Mean corpuscular hemoglobin, MCHC, Mean corpuscular hemoglobin concentration, PLT, Platelets, WBCs, White blood cells, SGPT, Serum glutamic pyruvic transaminase, HDL, High-density lipoprotein, LDH, Lactate dehydrogenase, SGOT, Serum glutamic-oxaloacetic transaminase, *Cyprinus carpio*, Heavy metal, Chromium, Manganese, Bioaccumulation

## Abstract

The present research work was carried out to determine the bioaccumulation of manganese and chromium in the gills, intestine, muscles, skin and bones, as well as its acute toxicity and effects on hematological and biochemical parameters in Common carp (*Cyprinus carpio*). Adult carps were exposed for 96 h to manganese sulphate and chromium chloride solution, a sub lethal concentration was used in the experiment. Bioaccumulation was highest in the gills followed by intestine > muscles > skin > bones. The concentration of hematocrit (HCT) (37.3 ± 0.36), hemoglobin (HGB) (9.0 ± 0.04), Red Blood Cells (RBCs) (3.7 ± 0.025), mean corpuscular volume (MCV) (121.2 ± 0.36), mean corpuscular hemoglobin (MCH) (41.3 ± 0.3) and mean corpuscular hemoglobin concentration (MCHC) (41.06 ± 0.072) was significantly higher at 96 h (P < 0.01) after exposure to manganese and chromium, while the concentration of platelets (PLT) (16.8 ± 0.12) and white blood cells (WBCs) (62.7 ± 0.11) was lower at 96 h of exposure. Serum glutamic pyruvic transaminase (SGPT) (40.6 ± 0.4), Blood Urea (13 ± 0.1), serum triglycerides (231.21 ± 0.04), high-density lipoprotein (HDL) (39 ± 0.07), serum Alkaline PO_4_ (242 ± 0.2), lactate dehydrogenase (LDH) (1239 ± 13.21), and serum Uric Acid (4.81 ± 0.33) were significantly higher (P < 0.01) at 96 h of exposure. The highest concentration of serum cholesterol (339 ± 0.09), serum reatinine (0.9 ± 0.01), low density lipid (240 ± 0.2) was observed at 24 h. Serum glutamic-oxaloacetic transaminase (SGOT) (19 ± 0.13), and serum albumin were at the highest level at 72 h (3.19 ± 0.07) (P < 0.01) post exposure.

## Highlights

• Heavy metals effects fishes when its concentration arises from its normal.
• Bioaccumulation of manganese and chromium was studied in *Cyprinus carpio* on hematological and biochemical parameters.
• In organ, bioaccumulation is highest in the gills followed by intestine > muscles > skin > bones.
• It is concluded that heavy metal can readily bio accumulate in the organs of fish.

## Graphical abstract

**Figure:**
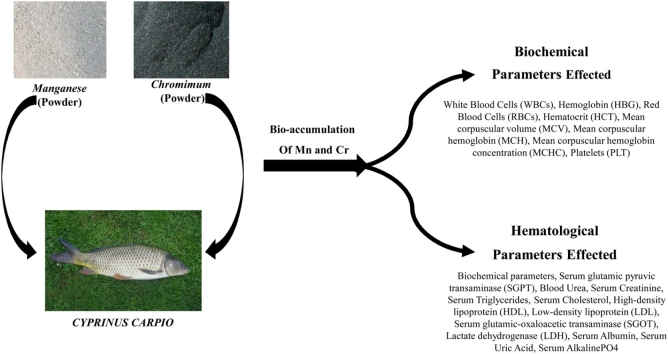

## 1 Introduction

Fishery makes significant contribution to the field of nutrition and trade as well, provided opportunities in employment, millions of people are doing their jobs in fishery and earn money to look after their families well so fishery also provide jobs for the people and play a role in the employments sector of a country [[1], [2], [3]].

Heavy metal pollution is an environmental problem of global concern, which often has ecological consequences threatening aquatic organisms. Heavy metals accumulate in skin, gills, intestine, liver, kidney and other organs of fish and causes physical as well internal damage to the fish body [[4], [5], [6]]. The study of hematological indices is useful in the diagnosis of many diseases and in the investigation of extent and damaged blood cells by toxic effects of different chemical or microbial effects [7,8].

The stressed behavior, irregular swimming patterns, hyperactivity and aggression, are consequences of environmental stress [9]. Overexposure to Mn2+ may have negative physiological effects on fish and other organisms inhabiting heavy metal polluted waters [10,11], found the highest bioaccumulation capacity in terms of Ca, Mg, Na, Ni, As, Zn and Cd was registered in caudal fin, liver and intestine tissues while K, Fe, Cu and Mn had the highest bioaccumulation in their muscle, spleen, liver and gills. Selenium (Se) is also toxic for aquatic organisms when present at high concentrations [12].

Hematological and biochemical parameters are important in diagnosing the structural and functional status of fish exposed to toxicants [[13], [14], [15]], describe xenobiotic molecules as the changer of physiological homeostasis of fish that can produce an oxidative stress. Differences in hematological parameters hematocrit, hemoglobin concentration, leukocyte and erythrocyte count have been used as pollution and physiological indicators of organic dysfunction in both environmental and aquaculture studies [[16], [17], [18]]. Salinity and seasonal variations can have an influence on the level of erythrocyte, hemoglobin, hematocrit, leucocytes and thrombocytes and on all biochemical parameters [19,16,20]. *C. carpio* is a common edible fish used among the world population therefore different experiments regarding toxicity of heavy metals have already been done which indicated that heavy metals directly affects fish health and can cause damages to its population. This design was intended to quantify the influence of manganese and chromium bioaccumulation in *C. carpio*, various tissues such as gills, intestine, muscles, skin and bones by examining hematological and biochemical parameters. This evaluation will also contribute to an upgraded knowledge of heavy metals bioaccumulation and its effect on *C. carpio*.

## 2 Material and methods

### 2.1 Ethical approval

Ethical approval of the study was given by 20th Advance Study Research Board (ASRB) meeting under item No.2; section 8(iv) of Islamia College Peshawar on 21 February 2019.

### 2.2 Fish collection and acclimatization

Total of 50 Adult *C. carpio* were obtained from Sherabad Carp hatchery District Peshawar, Khyber Pakhtunkhwa (KP) Pakistan, placed in a shopping bag filled with water, brought to the laboratory and were acclimatized for almost 3 weeks providing fresh water and 2% of food by weight on daily basis. After acclimatization, fishes were checked for mortality, 2 fishes were found dead and 48 were found fresh and healthy, free from any kind of disease causing agents or death precursors and ready to perform experiments. Before the experiment the length and weight of experimental carps were measured the average length and weight were 14 cm and 360 g respectively.

### 2.3 Preparation of stock solution for manganese sulphate and chromium chloride

Manganese sulphate and Chromuim chloride solution was used as test solution for the experiments. A stock solution 1000 mg/l (1000 ppm) of MnSO_4_ and CrCl_3_ were prepared by adding 1 g of Manganese sulphate and chromium chloride to 1 L of distal water and that was stored in a glass bottle. Sub-lethal concentrations, 1.12 mg/l of MnSO_4_ and 3.41 mg/l of CrCl_3_ were used based on the 96 h LC50 value for MnSO4 and CrCl3 i.e. 5.6 mg/l. and 17.05 mg/l respectively.

### 2.4 Experimental design

After acclimatization, the 96 h LC50 for MnSO4 was determined as 5.6 mg/l, and for of CrCl3 as 17.05 mg/l. Fish were exposed to two sub lethal concentrations of MnSO4 i.e. 1.12 mg/l, and CrCl3 i.e. 3.41 (20%, respectively of LC50 value). The carps were randomly distributed in three different glass tanks with a density of 16 fish per tank having 120 L of water. One tank was labeled as control group and the other two were labeled as treated groups. i.e. (Mn treated) and (Cr treated). Treated tanks were then exposed to the concentration of 1.12 mg/l for MnSO_4_ and 3.41 mg/l for CrCl_3_. No chemicals were added to the control group. After 24 h, four fish from each treated and control tank were sacrificed and dissected for the removal of different organs and analysis of bioaccumulation. Blood was collected for biochemical and hematological parameters. The same procedure was performed for 48 h, 72 h and 96 h four fish were sacrificed each day from each tank respectively.

### 2.5 Measurement and analysis of bioaccumulation

*C. carpio* were dissected and different visceral and body organs were isolated, about 0.5 g of tissue was cut off from Gills, intestine, muscles, skin and bones and kept in 10 mL of nitric acid for 24 h to be digested. After 24 h, for complete digestion samples were placed in a 100 °C Hot plate (Gallenkamp: A England, CAT No: SS260, APP No: 4-SS260, 6.5 Amp, 220/240 V) and then cooled down at room temperature adding 30 mL of distilled water and filtered by whatman filter paper. The filtrate was to be analyzed for the presence of heavy metals. For detection of Manganese and Chromium in different organs of fish atomic absorption spectrophotometer (Model: Analyst 700, Parkin Elmer, USA, Serial No: 700S5040102) was used.

### 2.6 Determination of LoD and LoQ

The limit of detection (LOD) was predicted from three times the standard deviation (SD) of ten replicates of the blank divided by the slope of the calibration curve. The limit of quantification (LOQ) was calculated from ten times the SD of ten replicates of the blank divided by the slope of the calibration curve [21].

### 2.7 Hematological and biochemical analysis

The blood samples were taken from the caudal vein of the fish by a sterile syringe containing heparin (1000 IU/mL) anticoagulant solution. The blood plasma was obtained by centrifugation of the blood at 3000 rpm for 15 min while the non-hemolyzed plasma was stored in a cool place for further biochemical observations. These blood samples were used for RBCs count following the method of [22], and the hemoglobin content [23]. The value of hematocrit, was calculated by the mentioned rules and formulae of [24], plasma glucose was determined by using assay kits supplied by Human Diagnostics Worldwide according to [25]. Total protein content was determined according to the method of [26] and lipid contents was determined by colorimetrically as by [27]. The activity levels of aspartate aminotransferase (AST) and alanine aminotransferase (ALT) were determined colorimetrically according to [28].

### 2.8 Statistical analysis

Graph pad prism version 6.01 was used for statistical analysis. ANOVA technique was used for the statistical analysis, means were separated according to the Fisher’s LSD (least significant difference) test and compared by using the Duncan’s Multiple Range test (DMRT). The Significant differences were defined at (*P* < 0.01)

## 3 Results and discussion

*C. carpio* is the common culture able fish species, exposing it to different concentrations of manganese and chromium results in high bioaccumulation in the gills. The toxicity of heavy metals is different in test organisms due to different mechanisms of action, chemical characteristics of test solution, sensitivity and tolerance limit of the test organism [[29], [30], [31]] noted that metal accumulation depends upon species, location and seasonality, with Seabass having higher heavy metal concentrations than seabream. Generally heavy metals accumulate in the metabolically active tissues of the body of living organisms [32] which is observed in the current study.

Heavy metals are elements with high density such as, Aluminum (Al), Arsenic (As), Cobalt (Co), Chromium (Cr), Copper (Cu), Iron (Fe), Magnesium (Mg), Manganese (Mn), Lead (Pb), Tin (Sn), Zinc (Zn) are quite toxic in low concentrations and can accommodate in different organs of fish [33,34]. Even trace amounts of heavy metal can be toxic to fish, and its toxicity is dependent on the concentration of heavy metal and it its most bioavailable form [35]. Chromium and its particulates enter the aquatic medium from different industries such textiles, electroplating workshops, dyeing, and medical industries, as it is a commonly used metal. The most toxic form is hexavalent chromium it can readily cross cellular membranes and then reduced to trivalent form. This trivalent chromium combines with several macromolecules including genetic material inside the cytosol, and ultimately alters behavior, physiology, cytology, histology and morphology [36].

The limits of detection (LOD) and the limits of quantification (LOQ) in present study were calculated based on the standard deviation of 10 readings obtained for the analytical blanks and the slopes of the analytical curves. The values (mg/kg) were 0.042–0.078 (Mn) and 0.062–0.153 (Cr).

**Fig. 1:**
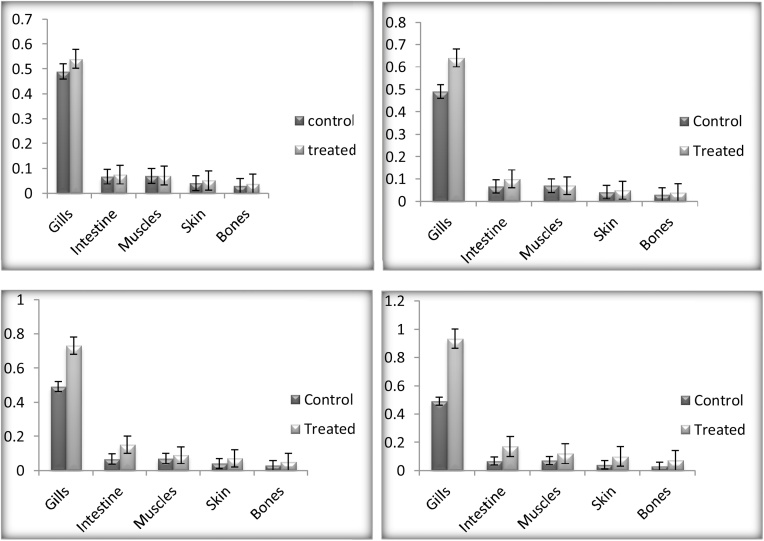
Showing manganese bioaccumulation (μg/g dry weight of fish) in gills, intestine, muscles, skin and bones of both control and treated *C. carpio*, exposed to manganese sulphate for 24, 48, 72 and 96 h respectively.

**Fig. 2:**
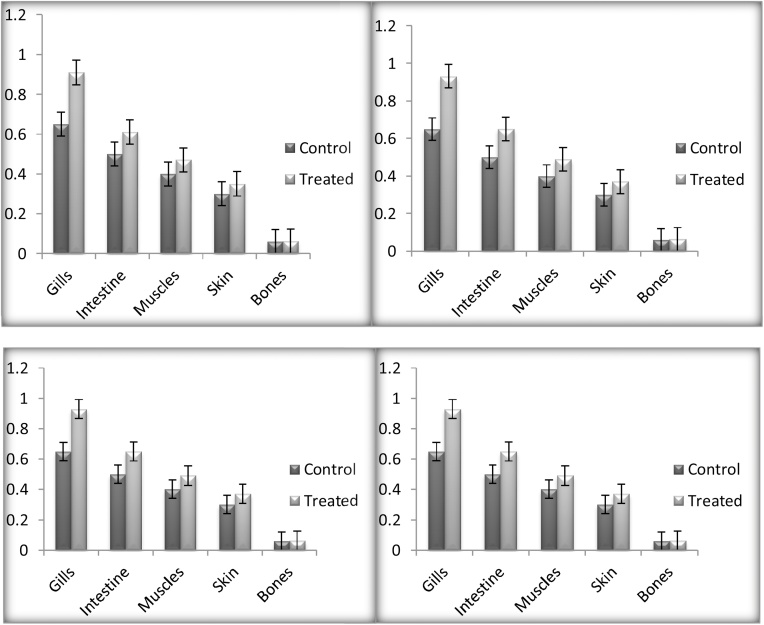
Showing chromium bioaccumulation (μg/g dry weight of fish) in gills, intestine, muscles, skin and bones of both control and treated *C. carpio*, exposed to chromium chloride for 24, 48, 72 and 96 h respectively.

After 24 h of exposure the Mn and Cr (0.54 ± 0.04) and (0.78 ± 0.01) was highly detected in the gills of the *C. carpio* followed by intestine, while significantly low accumulation was detected in the bones. [37] detect high concentrations of chromium (570 ± 52.1) and manganese (66.7 ± 8.5) in the gills of *C. carpio* collected from river Kabul. Muscles and skin also have low concentration compared to gills and intestine. Gills are the first target and directly exposed to the water-born heavy metals [38,39]. Gill surface is negatively charged and has the potential for the positive charged metals [[40], [41], [42]]. Fish which take heavy metals in their feed have maximum and elevated levels of heavy metal in the digestive tract as compared to their gills [43,44]. Skin is in direct contact with the external environment and that also results in elevated levels of heavy metals [37]. Similar trends were observed after 48 h bioaccumulation, gills accumulated high concentrations (0.64 ± 0.07) and (0.79 ± 0.19) compared to 24 h of exposure, muscles and intestine followed the same trend of accumulation as 24 h of exposure. While exposing *C. carpio* up to 72 h the overall accumulation was considerably high (0.73 ± 0.07) and (0.8 ± 0.21) in the organs compared to 48 h. Moreover, during 96 h of exposure gills accumulated a high concentration of Mn (0.933 ± 0.08) and Cr (0.8 ± 0.24) compared to 72 h. The pattern of bioaccumulation of heavy metals versus time of exposure followed pattern 96 h > 72 h > 48 h > 24 h (P < 0.01) while the accumulation in organs are in sequence like gills > intestine > muscles > skin > bones. Figs. 1 and 2 showing concentrations of manganese and chromium in different organs of the treated organisms. Concentration of heavy metals detected after different time exposure shown in Table 1.

**Table 1:** Showing bioaccumulation in the gills, intestine, muscles, skin and bones after 24, 48, 72 and 96 h exposure of *C. carpio* to manganese and chromium. All the values are expressed as (Mean ± SE) using Fisher’s LSD test. Presented values are Significant (≥0.1) at *p* ≤ 0.01.

Time of Exposure	Organs	Manganese		Chromium	
		Control	Treated	Control	Treated
24 h	Gills	0.49 ± 0.07	0.54 ± 0.04	0.65 ± 0.18	0.78 ± 0.01
	Intestine	0.067 ± 0.04	0.07 ± 0.09	0.5 ± 0.07	0.5 ± 0.01
	Muscles	0.07 ± 0.04	0.07 ± 0.07	0.4 ± 0.04	0.43 ± 0.02
	Skin	0.041 ± 0.04	0.05 ± 0.07	0.3 ± 0.03	0.32 ± 0.01
	Bones	0.03 ± 0.04	0.03 ± 0.07	0.06 ± 0.07	0.06 ± 0.05
48 h	Gills	0.49 ± 0.07	0.64 ± 0.07	0.65 ± 0.18	0.79 ± 0.19
	Intestine	0.067 ± 0.04	0.1 ± 0.04	0.5 ± 0.07	0.57 ± 0.10
	Muscles	0.07 ± 0.04	0.07 ± 0.04	0.4 ± 0.04	0.45 ± 0.06
	Skin	0.041 ± 0.04	0.05 ± 0.04	0.3 ± 0.03	0.33 ± 0.04
	Bones	0.03 ± 0.04	0.04 ± 0.07	0.06 ± 0.07	0.06 ± 0.02
72 h	Gills	0.49 ± 0.07	0.73 ± 0.07	0.65 ± 0.18	0.8 ± 0.21
	Intestine	0.067 ± 0.04	0.159 ± 0.04	0.5 ± 0.07	0.58 ± 0.12
	Muscles	0.07 ± 0.04	0.09 ± 0.04	0.4 ± 0.04	0.45 ± 0.07
	Skin	0.041 ± 0.04	0.07 ± 0.1	0.3 ± 0.03	0.34 ± 0.06
	Bones	0.03 ± 0.04	0.05 ± 0.01	0.06 ± 0.07	0.06 ± 0.03
96 h	Gills	0.49 ± 0.07	0.933 ± 0.08	0.65 ± 0.18	0.8 ± 0.24
	Intestine	0.067 ± 0.04	0.177 ± 0.09	0.5 ± 0.07	0.6 ± 0.14
	Muscles	0.07 ± 0.04	0.12 ± 0.04	0.4 ± 0.04	0.45 ± 0.07
	Skin	0.041 ± 0.04	0.1 ± 0.04	0.3 ± 0.03	0.35 ± 0.07
	Bones	0.03 ± 0.04	0.07 ± 0.04	0.06 ± 0.07	0.06 ± 0.04

### 3.1 Hematological indices

Chromium and manganese is absorbed into the fish from the water and both of them interfere and alter the hematological and biochemical parameters of fish blood [45,46]. All the heavy metals induces increase in the frequency of erythroblast cells which was particularly in the Pb exposed fish, this shows the stress related to the catecholamine-induced contraction of the spleen where the blood cells stores, and the within a short interval of time it releases new erythrocytes cells to the bloodstream [47] but [48] reports that the quantitative red blood parameters are rather stable and little sensitive to environmental factors, due to considerable compensatory abilities of fish organism. Hematology is the best indicator to express the health status of fish, exposing *C. carpio* to heavy metals can bring prominent change in the hematological indices of fish, similar change was also observed in the present study, the concentration of hematocrit (HCT) (41.1 ± 0.21), hemoglobin (HGB) (12.9 ± 0.11), red blood cells (RBCs) (3.8 ± 0.32), mean corpuscular volume (MCV) (143.5 ± 1.4), mean corpuscular hemoglobin (MCH) (47.6 ± 0.3), procalcitonin blood test (PCT) (0.037 ± 0.01) and mean corpuscular hemoglobin concentration (MCHC) (47.1 ± 0.4) was significantly high at 96 h (*P* < 0.01) after exposure to Manganese and chromium, while the concentration of platelets (PLT) (12.4 ± 0.13) and white blood cells (WBCs) (39 ± 0.9) was considerably low at 96 h of exposure while high at 24 h (*P* < 0.01) shown in Table 2.

**Table 2:** Showing hematological parameters of both control and treated *C. carpio* after exposure time of 24, 48, 72 and 96 h to combine effect of manganese and chromium. All the values are expressed as (Mean ± SE) using Fisher’s LSD test. Presented values are Significant (≥0.1) at *p* ≤ 0.01.

	Control	Treated			
Hematological Indices		24 h	48 h	72 h	96 h
White Blood Cells (WBCs)	115 ± 1.3	66.2 ± 0.1	47 ± 0.33	45 ± 0.450	39 ± 0.9
Hemoglobin (HBG)	12.5 ± 0.5	12.9 ± 0.7	12.1 ± 0.34	11.4 ± 0.7	12.9 ± 0.11
Red Blood Cells (RBCs)	2.4 ± 0.4	2.8 ± 0.7	3.09 ± 0.11	3.1 ± 0.9	3.8 ± 0.32
Hematocrit (HCT)	29.5 ± 0.3	31.05 ± 07	33 ± 0.12	35.5 ± 0.11	41.1 ± 0.21
Mean corpuscular volume (MCV)	107 ± 1.4	112 ± 0.07	133 ± 0.3	131.5 ± 0.3	143.5 ± 0.4
Mean corpuscular hemoglobin (MCH)	33.01 ± 0.5	40 ± 0.2	43 ± 0.15	43.5 ± 0.13	47.6 ± 0.3
Mean corpuscular hemoglobin concentration (MCHC)	28.1 ± 0.9	30.1 ± 0.09	41.2 ± 0.34	45.5 ± 0.6	47.1 ± 0.4
Platelets (PLT)	18 ± 0.7	18 ± 0.1	19 ± 0.16	13 ± 0.9	12.4 ± 0.13

### 3.2 Biochemical parameters

Variations in fish proteins can be used as a bio-indicator to monitor the physiological status of the treated fish [49]. Inhibited or elevated enzyme activity compared to reference groups serves as a diagnostic tool in toxicology and is a good marker of metabolic changes in fish, i.e., hypoxic conditions, impaired antioxidant mechanisms, and cellular or tissue damage in fish [50]. During the present study exposing *C. carpio* to heavy metals (Mn, Cr) significant difference was observed in level of biochemical parameters, i.e level of serum glutamic pyruvic transaminase (SGPT) (40.6 ± 0.49) was significantly high (*P* < 0.01) at 96 h of exposure to heavy metals while low value was noticed at 24 h (23.5 ± 0.23), similar trend was followed by Blood Urea (13 ± 0.1), Serum Creatinine (0.21 ± 0.36), high-density lipoprotein (HDL) (39 ± 0.07), Serum Alkaline PO4 (242 ± 0.2). Serum triglycerides were significantly low (231.21 ± 0.04) at 24 h (*P* < 0.01) while high (239.2 ± 0.04) at 96 h of exposure. Highest values of Serum Cholesterol (339.06 ± 0.098) and low density lipid (LDL) (240.1 ± 0.15) were detected at 24 h. Serum glutamic-oxaloacetic transaminase (SGOT) was high at 72 h (19 ± 0.13), lactate dehydrogenase (LDH) was significantly high at 96 h (1239 ± 13.21) (*P* < 0.01), moreover Serum Albumin was low at 24 h (2.7 ± 0.02) while high at 72 h (3.09 ± 0.04), Serum Uric Acid was considerably low (4.09 ± 0.04) at 24 h and high (4.81 ± 0.03) at 96 h (*P* < 0.01) while all the values of control groups were low compared to treated show in Table 3. Aspartate aminotransferase (AST) and alanine aminotransferase (ALT) are liver specific enzymes that are a more sensitive measure of hepatotoxicity and histo-pathological changes and can be assessed within a shorter time. The marked increase in the level of AST, showed liver dysfunction [51]. Due to increasing exposure time and concentration of heavy metals, the level of bioaccumulation in the *C. carpio* increases accordingly, gills the direct exposed organs accumulated high concentration compared to other organs. The overall results from the present research work shows that excess amount of heavy metals affect the physiological, biochemical and hematological parameters of the fish and can affect the fish growth and normal body functions.

**Table 3:** Showing biochemical parameters of both control and treated *C. carpio* after exposure time of 24, 48, 72 and 96 h to combine effect of manganese and chromium. All the values are expressed as (Mean ± SE) using Fisher’s LSD test. Presented values are Significant (≥0.1) at *p* ≤ 0.01.

	Control	Treated				
Biochemical parameters			24 h	48 h	72 h	96 h
Serum glutamic pyruvic transaminase (SGPT)	29 ± 0.3		23.5 ± 0.2	37 ± 0.09	39 ± 0.13	40 ± 0.4
Blood Urea	9 ± 0.07		11 ± 0.9	9 ± 0.09	13 ± 0.04	13 ± 0.1
Serum Creatinine	0.9 ± 0.01		0.4 ± 0.2	0.14 ± 0.02	0.18 ± 0.05	0.21 ± 0.36
Serum Triglycerides	204 ± 4.1		231 ± 0.04	2 18 ± 0.1	221 ± 0.1	239 ± 0.04
Serum Cholesterol	189 ± 2.31		339 ± 0.09	202 ± 0.21	205 ± 0.33	189 ± 0.2
High-density lipoprotein (HDL)	26 ± 0.31		37 ± 0.2	36 ± 0.17	39 ± 0.31	39 ± 0.07
Low-density lipoprotein (LDL)	124 ± 2.1		240 ± 0.2	139 ± 0.21	139 ± 0.11	124 ± 0.2
Serum glutamic-oxaloacetic transaminase (SGOT)	10 ± 06		13 ± 0.1	16 ± 0.7	19 ± 0.13	8.3 ± 0.1
Lactate dehydrogenase (LDH)	1118 ± 11.1		1230 ± 0.1	1227 ± 0.1	1136 ± 0.1	1239 ± 0.21
Serum Albumin	1.6 ± 0.8		2.7 ± 0.1	2.9 ± 0.3	3.1 ± 0.7	3.09 ± 0.04
Serum Uric Acid	1.7 ± 0.1		4.09 ± 0.02	3.2 ± 0.7	3.3 ± 0.14	4.8 ± 0.03
Serum AlkalinePO4	194 ± 4.21		198 ± 0.2	210 ± 0.15	214 ± 0.2	242 ± 0.2

## 4 Conclusion

Present results show that manganese and chromium accumulated in different organs of the fish. Highest bioaccumulation was observed in gills while lowest in bones. Intestines also accumulate high concentration of manganese and chromium due to dietary heavy metals. Current results show that the heavy metal not only leads to bioaccumulation but also severely affects the fish biochemistry and hematology. It is suggested, to further evaluate the effect of heavy metals on other fish species and its impact on human health.

## Availability of data and materials

The datasets used and analyzed during the current study are available from the corresponding author on reasonable request.

## Author statement

Zeeshan Ali, Ali Muhammad Yousafzai and Nadia Sher collected the fishes for the study, performed experiments and written initial draft; Ijaz Muhammad, Gul E Nayab, Syed Abdul Maajid Aqeel; Syed Touheed Shah and Ijaz Khan helped in the hematological studies and statistical analysis. Haroon Khan has designed and supervised the overall study.

## Authors contribution

AMY designed and supervised the study. ZA, NS, IM, GN, SAMA, STS and IK collected samples and conducted experiments. ZA, IM, HK and GN draft the manuscript. All authors read and approved the manuscript.

## Funding

No funding source. The study is self-supported.

## Declaration of Competing Interest

The authors report no declarations of interest.
